# Dexamethasone-Loaded Ureasil Hydrophobic Membrane for Bone Guided Regeneration

**DOI:** 10.3390/pharmaceutics14051027

**Published:** 2022-05-10

**Authors:** Rafaella Moreno Barros, Camila Garcia Da Silva, Kammila Martins Nicolau Costa, Arnóbio A. Da Silva-Junior, Cássio Rocha Scardueli, Rosemary Adriana Chiérici Marcantonio, Leila Aparecida Chiavacci, João Augusto Oshiro-Junior

**Affiliations:** 1 Pharmaceutical Sciences Postgraduate Center for Biological and Health Sciences, State University of Paraíba, Av. Juvêncio Arruda, S/N, Campina Grande 58429-600, Paraíba, Brazil; rafaellamoreno@yahoo.com.br (R.M.B.); kammilamartiins@hotmail.com (K.M.N.C.); 2 Department of Drugs and Medicines, School of Pharmaceutical Sciences, São Paulo State University (UNESP), Highway Araraquara-Jaú, Araraquara 14801-902, São Paulo, Brazil; mila_garcia1@hotmail.com; 3 Department of Pharmacy, Federal University of Rio Grande do Norte-UFRN, Natal 59012-570, Rio Grande do Norte, Brazil; arnobiosilva@gmail.com; 4 Faculty of Dentistry, São Paulo State University (UNESP), Araraquara 14801-385, São Paulo, Brazil; cassiorochas@hotmail.com (C.R.S.); adriana.marcantonio@unesp.br (R.A.C.M.)

**Keywords:** critical bone defect size, hydrophobic membrane, organic–inorganic hybrid materials

## Abstract

Physical barrier membranes have been used to release active substances to treat critical bone defects; however, hydrophilic membranes do not present a prolonged release capacity. In this sense, hydrophobic membranes have been tested. Thus, this study aimed to develop hydrophobic membranes based on mixtures of ureasil–polyether-type materials containing incorporated dexamethasone (DMA) for the application in guided bone regeneration. The physicochemical characterization and biological assays were carried out using small-angle X-ray scattering (SAXS), an in vitro DMA release study, atomic force microscopy (AFM), a hemolysis test, and in vivo bone formation. The swelling degree, SAXS, and release results revealed that the u-PPO400/2000 membrane in the proportion of 70:30 showed swelling (4.69% ± 0.22) similar to the proportions 90:10 and 80:20, and lower than the proportion 60:40 (6.38% ± 0.49); however, an equal release percentage after 134 h was observed between the proportions 70:30 and 60:40. All u-PPO materials presented hemocompatibility (hemolysis ≤2.8%). AFM results showed that the treatments with or without DMA did not present significant differences, revealing a flat/smooth surface, with no pores and/or crystalline precipitates. Finally, in vivo results revealed that for both the commercial hydrophilic membrane and u-PPO400/2000 (70:30) after 60 days, the bone formation volume was 21%. In conclusion, hybrid membranes present unique characteristics for treating critical bone defects, considering the delayed and prolonged release results associated with the physical barrier capacity.

## 1. Introduction

The guided bone regeneration technique is a procedure that has the characteristic of applying a barrier membrane to cover the bone defect area. This is aimed to prevent the migration of cells that interfere with bone formation, creating an environment with ideal conditions for tissue regeneration. Since the 1950s, novel biomaterials have been studied for these applications [1,2,3].

Dexamethasone (DMA) has been used in the postoperative period to overcome immune reactions [4]. DMA is one of the most effective steroidal anti-inflammatory drugs, with the solution and tablet pharmaceutical forms being the most commonly used orally. However, it has disadvantages due to its hydrophobicity, lack of selectivity, and low bioavailability, requiring high doses to reach the therapeutic level. Therefore, it may result in undesirable side effects, such as osteoporosis, increased blood sugar concentration, hypertension, and stomach and intestinal bleeding [5].

Different studies have already demonstrated the high incorporation capacity of DMA, which has been used in high and low concentrations and in different pharmaceutical forms, seeking to minimize side effects and at the same time, to have the capacity to achieve the proposed objective, such as bone tissue improvement, and inflammation and immunosuppressive effects [6,7]. Moreover, DMA acts on different pathways in osteogenic differentiation, such as the induction of Runx2 expression by FHL2/β-catenin-mediated transcriptional activation, enhancing the bone healing process [8].

The clinical grafting technique is currently associated with a membrane by using a procedure known as guided bone regeneration, which has a physical barrier capacity and is added to the surgical site. In this sense, an attractive therapeutic strategy is the incorporation of DMA into these membranes. This procedure is aimed to mitigate the postsurgical side effects of dexamethasone and the immunological reactions concerning the grafts [9,10,11].

However, the significant challenge from a technological perspective is finding a material with adequate characteristics concerning biocompatibility, mechanical properties, and biodegradation. These factors can either inhibit or benefit the healthy pattern of bone tissue formation [10].

Currently, on the market, membranes are composed of substances such as collagen, chitosan, and polyethylene glycol [12]. Thus, commercially, no material can meet the appropriate characteristics and at the same time, control and release the active substances such as DMA [13].

In this context, a class of materials that has been gaining prominence by presenting biocompatibility, flexibility, and sustained release capacity are the organic–inorganic type materials which present an excellent rate of in vivo and in vitro activity. Additionally, they can be developed with polymers or a mixture of polymers with hydrophobic characteristics, providing the possibility of release for weeks, which is essential for the formation of bone cells and periodontal ligaments, and even months (in the case of large defects), which is necessary for perfect bone formation and maturation [12,14].

This work aimed to develop a hydrophobic hybrid membrane containing DMA, evaluating its physicochemical characteristics and carrying out preclinical studies in animals in order to develop a membrane with dual functionality as a physical barrier and a drug release matrix.

## 2. Materials and Methods

### 2.1. Preparation and Incorporation of API into Ureasil–Polyether Membranes

The synthesis of the hybrid percussor was carried out following the sol–gel chemical route technique. Initially, a modified alkoxide 3-isocyanatopropyltriethoxysilane (IsoTrEOS) (Sigma-Aldrich, São Paulo, Brazil) and two modified polymers (NH_2_-PPO-NH_2_) (Sigma-Aldrich, São Paulo, Brazil), with different molecular masses of 400 and 2000 g/mol, were reacted in tetrahydrofuran (THF) (Sigma-Aldrich, São Paulo, SP, Brazil) (polymer/alcohol molar ratio 1:2; kept under reflux for 24 h at 60 °C). THF was removed by evaporation, forming the hybrid precursor (EtO)_3_Si(CH_2_)_3_NHC(=O)NHCHCH_3_CH_2_(polyether)CH_2_CH_3_CHNH(O=)NHC(CH_2_)_3_Si(OEt)_3_, named hereafter u-PPO400 and u-PPO2000.

**Figure 1:**
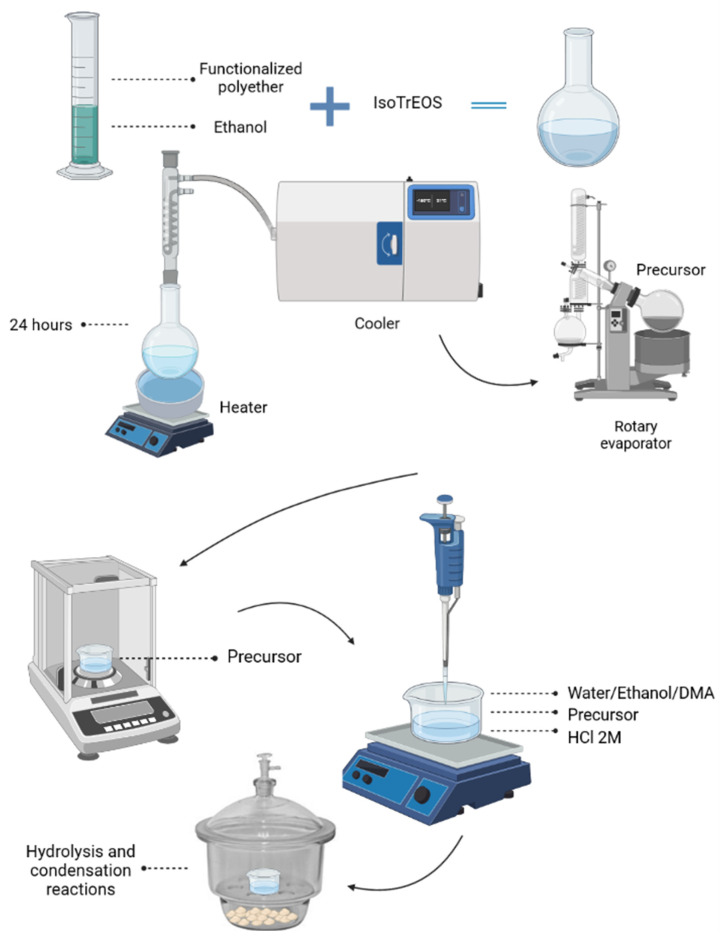
The representative scheme of the methodology steps applied to prepare the hybrids and incorporate dexamethasone. Image created with the assistance of BioRender.

Subsequently, this precursor was subjected to hydrolysis and condensation reactions. In this step, 500 µL of alcohol, 50 µL of water, and an acid catalyzing agent (HCl) at a concentration of 2 M were added. As the reactions proceeded, the progressive elimination of the OH groups and the formation of a gel occurred. Thus, the membranes were vacuum-dried for 24 h and cut into a disk shape with 6.0 mm diameter and 0.5 mm thickness. The entire process of preparing the hybrid precursors and incorporating the DMA can be observed from the schematic shown in Figure 1.

To evaluate the maximum concentration of the incorporated DMA, different masses of DMA (1, 3, 6, and 10% *w*/*w*) were added to 0.075 g of ureasil–polyether hybrid precursor during the hydrolysis and condensation stages.

### 2.2. Macroscopic Swelling

The membranes were inserted into a beaker containing 500 mL of phosphate buffer pH 7.2, at 37 °C (±0.5 °C) and stirred at 50 rpm. At pre-established time intervals (0, 0.25, 0.5, 1, 1.5, 2, 3, 4, 6, 12, 48, and 72 h), the membranes were removed, dried, and mass measurements were made using an analytical balance. The percentage of water absorbed by the membranes was calculated by the difference in weight before and after it was immersed in saliva. The results were presented as the mean ± standard deviation (SD) of triplicates.

### 2.3. Small-Angle X-ray Scattering (SAXS)

The experiment was performed in the SAXS synchrotron light line laboratory at LNLS (Campinas, São Paulo, Brazil). Its structure is composed of an asymmetrically cut and curved Si (111) monochromator that produces a horizontally focused beam (λ = 0.1608 nm). A vertical position-sensitive X-ray detector and a multichannel analyzer were used to record the SAXS intensity, I (*q*), as a function of the modulus of the scattering vector *q* = (4πλ) sin (ε/2), with ε being the scattering angle. The samples were analyzed under stable temperature and pressure, with SAXS patterns being recorded every 30 s.

### 2.4. Analytical Curve

From an ethanolic solution of DMA (Sigma-Aldrich, São Paulo, Brazil) (100 µg/mL), the following dilutions were prepared: 1, 5, 10, 20, 30, 40, 45, and 50 µg/mL. The analyses were performed in the AGILENT CARY 60 ultraviolet spectrophotometer at a wavelength (λ) of 241 nm and the calibration curve was constructed (analyte concentration × absorbance).

### 2.5. In Vitro DMA Release

The membranes were submitted to in vitro dissolution tests in phosphate buffer solution under the following conditions: pH 7.2; 0.5% (*v*/*v*) of procetyl; 37 °C; 500 mL of phosphate buffer solution at constant 50 rpm agitation (AGILENT 108-DS dissolution apparatus). After the predetermined time intervals (0.25, 0.50, 0.75, 1, 2, 3, 4, 6, 12, 24, 48, 72, 96, 120, and 144 h), using a syringe (7.75 AGILENT edge cannula), aliquots (5 mL) were collected and filtered through a 0.45 µm pore size filter (AIICROM Filters). The collected volume of the aliquots was then replenished.

The spectra were analyzed in an AGILENT CARY 60 ultraviolet spectrophotometer at a wavelength (λ) of 241 nm. The test was performed in triplicate.

### 2.6. Atomic Force Microscopy

Topography measurements of the ureasil–polyether hybrid systems were performed with and without the use of the drug. The analysis was carried out using an atomic force microscope (Bruker, Dimension Icon model, Santa Barbara CA, USA) under the intermittent contact mode. The sample was added to the microscope, and then topographical images were observed by employing a probe/cantilever that traced the sample’s surface. The treatment of the topographic images was performed using Nanotec WSxM software version 5.0 (Madrid, Spain) [15].

### 2.7. Evaluation of the Hemolytic Potential of Ureasil–Polyether Materials

The test followed the methodology recommended by the American Society for Testing and Materials—ASTM (ASTM F756), with modifications. After approval by the Ethics Committee of the Universidade Estadual da Paraíba—UEPB (CAAE 52812121.1.0000.5187), volunteer adult individuals of both sexes, aged between 18 and 50 years old, carriers of blood types A, B, and O were recruited, and blood samples were collected in EDTA tubes. Subsequently, the blood samples were centrifuged (Hettich^®^—MIKRO 220 R centrifuge (2000 rpm for 5 min)) to separate the plasma, which was discarded. A 0.9% NaCl solution was added at a proportion of 1:1 (*v*/*v*) followed by centrifugation. The procedure was performed twice to obtain a red blood cell sediment. Finally, an aliquot was removed and added to a new test tube with 0.9% NaCl to obtain a 0.5% blood suspension (0.5 mL of the suspension in 99.5 mL of 0.9% saline solution).

The ureasil–PPO400/2000 samples in different proportions were cut into 1 cm² and incubated in 7 mL of 0.9% saline solution (37 °C for 24 h). The saline solution was removed, and 50 µL of the red cell concentrate was added to the surface of the test sample, which remained in contact for 15 min. After this time, 10 mL of 0.9% saline solution was added (3 h incubation at 37 °C). Once incubation was complete, the fluid was transferred to a test tube and centrifuged at 104 rpm for 15 min.

The hemoglobin released by hemolysis was measured by the absorbance of the supernatant at 545 nm using a UV–vis spectrophotometer—Shimadzu^®^ UV-1900 (Barueri, SP, Brazil). The following controls were used: a positive control, with 50 µL of the red cell suspension + 10 mL of deionized water, and a negative control, with 50 µL of the red cell suspension + 10 mL of 0.9% saline solution. The percentage of hemolysis was calculated by using Equation (1):

(1) $$\%Hemolysis=\frac{\left(Abs_{sample}-Abs_{neg.\;control}\right)}{\left(Abs_{pos.\;control}-Abs_{neg.\;control}\right)}\times 100$$

with:

*Abs_sample_* = absorbance of the sample;

*Abs_neg_._control_* = absorbance of the negative control;

*Abs_pos_._control_* = absorbance of the positive control.

### 2.8. In Vivo Assay

Thirty-six male rats (*Rattus norvegicus*, *Holtzman albinus* strain), aged 12 weeks and with a body mass between 250 g and 300 g, from the vivarium of the Faculdade de Odontologia de Araraquara (FOAr)—UNESP, were used. The protocol was approved by the local committee (Proc. Comissão de Ética no Uso de Animais—CEUA no. 17/2012, São Paulo, Brazil). Prior to and during the experiment, the animals were kept in a climate-controlled environment (22 °C), with a 12 h light cycle and access to water and feed ad libitum.

The animals were subdivided into two groups, four periods and six animals per period (6 *n*). In the preoperative stage, the animals were administered with anesthesia by an association of the APIs ketamine with xylazine (0.08 mL/100 g of ketamine hydrochloride 10% (Francotar, São Paulo, Brazil) and 0.04 mL/100 g of chloridrotoxilazine (Virabaxil 2%, São Paulo, Brazil). Subsequently, trichotomy was performed in the region of the skull cap and local asepsis (sterile gauze soaked in 10% povidone solution).

A semilunar incision (1.5 cm; interchangeable blade scalpel no. 15) was performed, then the subcutaneous tissue was dissected, allowing the periosteum exposure.

Subsequently, critical circular bone defects were made in the medial portion of the calvaria with 5 mm in diameter and approximately 1.5 mm thick. A trephine cutter (Dentoflex, São Paulo, Brazil) with 5 mm in outer diameter was used, mounted at a counter angle (Anthogyr-Injecta, Diadema, Brazil) with a 16:1 reduction, coupled to an implant motor (BLM 600 Plus Driller, Carapicuíba, Brazil), with 1500 rpm, and receiving constant irrigation of sterile saline solution to prevent thermal damage to the tissues. Lastly, the membranes were placed on the bone defect area, the incisions were sutured (Johnson 4-0 silk thread), and paracetamol analgesic (50 mg for each 100 g of body weight) was administered.

The membranes were randomly distributed to each animal (two bone defects) and divided into four experimental periods of 7, 15, 30, and 60 days, with six animals in each group/period.

The materials were divided into two groups. The first group, named GI, was composed of collagen membranes (Bio-Gide, Geistlich Pharma Ag-Biomaterials). The second, GII, corresponded to the group of ureasil–PPO400/2000 (70:30) membranes.

After the surgical procedure, were injected subcutaneously penicillin associated with streptomycin (Pentabiotic; FortDodge, Campinas, Brazil) and intramuscularly dipyrone sodium 5 mg/kg (Febrax; LemaInjex Biologic, São Paulo, Brazil) at 12 h intervals for 2 days.

After the four experimental periods, the animals were sacrificed through an overdose of anesthetic (ketamine and xylazine). Finally, a circular incision was made in the cranial skull using scissors and cutting pliers to remove the soft and hard tissues. These samples were fixed in 10% (*v*/*v*) buffered formalin for approximately ten days and then analyzed in the laboratory.

### 2.9. Three-Dimensional Radiographic Evaluation (Micro-CT)

The samples stored in 70% alcohol were submitted to an X-ray beam scanning analysis on a digital computerized microtomography system. The specimens were scanned using a microtomographer SkyScan 1176 Bruker MicroCT (Aatselaar, Belgium, 2003) by using 35 μm thick sections (50 kV and 500 μm), with a copper and aluminum filter and a rotation step of 0.3 mm. The images obtained via the projection of X-rays in the samples were stored and reconstructed, determining the area of interest by using NRecon software (Skyscan, 2011; version 1.6.6.0 (SkyScan, Konitich, Belgium)). In DataViewer software (SkyScan, version 1.4.4, 64-bit), the images were reconstructed for the observation in three planes (transverse, longitudinal, and sagittal). Then, with CTAnalyser—AWC software (2003-11SkyScan, 2012 Bruker Micro-CT Version 1.12.4.0 (Atibaia, São Paulo, Brazil), the area around the created bone defect was defined (region of interest: circular area with a diameter of 5 mm), and hence, the area of the bone formation was established. The threshold used in the analysis was 25–90 shades of gray.

### 2.10. Statistical Analysis

GraphPad software version 5.0 was used for statistical analysis (GraphPad Software, San Diego, CA, USA). All data were analyzed by an analysis of variance (ANOVA) and the mean values were compared by a post hoc Tukey test at the 5% significance level.

## 3. Results and Discussion

### 3.1. Preparation and Incorporation of DMA into Ureasil–Polyether Membranes

The initial stage of this study aimed to present the visual aspects of membranes developed with mixtures of ureasil–polyether precursors with molecular masses of 400 and 2000 g/mol. The mixtures were named u-PPO400/2000, and the proportions performed were 90:10, 80:20, 70:30, and 60:40. In addition, their homogeneity was analyzed when different proportions of DMA were incorporated.

**Figure 2:**
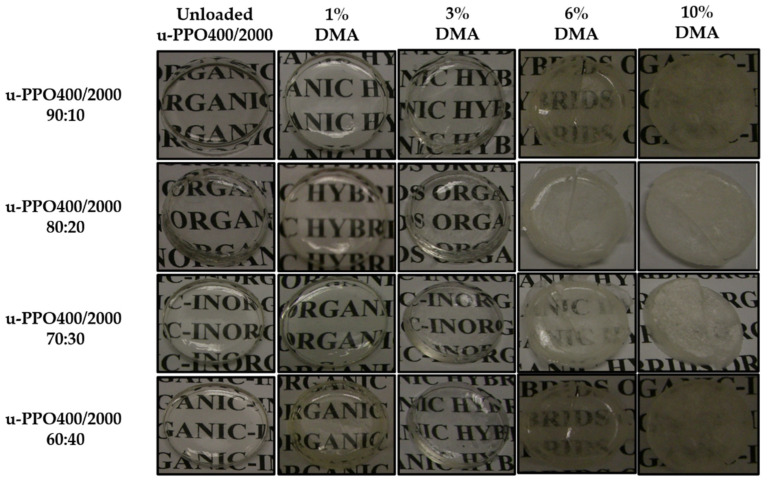
Visual characteristics of membranes from mixtures of the ureasil–polyether precursor: u-PPO400/2000 in the proportions of 90:10; 80:20; 70:30 and 60:40 without dexamethasone and containing 1, 3, 6 and 10% dexamethasone. Abbreviation: DMA = dexamethasone.

The visual appearance of the membranes with and without DMA is shown in Figure 2.

Regardless of the proportion of hybrid precursors, the u-PPO400/2000 membranes without DMA presented a crystalline aspect, with the absence of cracks, splits, and bubbles, and structural uniformity. However, these characteristics were modified with increasing DMA concentrations (>3%), as observed in Figure 2. At DMA concentrations of 6% and 10%, cracks and opacity were observed, in addition to the loss of uniformity due to the accumulation of DMA precipitated on the surface, demonstrating that the materials were not able to solubilize and trap the higher concentrations inside.

In the literature, different organic or inorganic materials isolated or in association have been studied for DMA incorporation to control the inflammation and improve osteogenic capacities, such as chitosan and chitosan/montmorillonite [16], RADA 16-I [7], photocross-linkable gelatin methacrylamide, and nanodiamonds [17] and PLGA/gelatin [18]; however, the DMA capacity of incorporation in these materials is limited, due to their insolubility in water. Thus, the hybrid materials, due to their chemical groups (R-Si-OH and CO(NH_2_)_2_), showed a high capacity of incorporation (3% *w*/*w*) and were chosen for further experiments.

### 3.2. Macroscopic Swelling

The swelling of a membrane reflects its ability to interact specifically with the absorbed molecules. The swelling test can be used to explain the release profile of the active pharmaceutical ingredient, and different factors can affect the results, such as the pH, temperature, swelling environment, nature of polymers, and degree of cross-linking [18,19,20].

Table 1 shows the swelling profile of the membranes obtained from the mixtures prepared with different concentrations of u-PPO400/2000.

The results presented indicate that although not very distinct, there was a statistically significant increase in the swelling of the membranes among the various proportions evaluated. It is possible to observe that as the proportion of the u-PPO2000 precursor (higher molecular weight) in the membrane increases, a higher swelling was observed, for instance, after 72 h, values of 4.32 ± 0.11, 4.58 ± 0.15, 4.69 ± 0.22, and 6.38 ± 0.49 were observed for the proportions of u-PPO400/2000 of 90:10, 80:20, 70:30, and 60:40, respectively.

However, despite the increase in swelling as a function of increasing u-PPO2000, these results were not pronounced. This fact is explained by both u-PPO400 and 2000 having CH_3_ groups in the ether-type oxygen, which gives the sample a hydrophobic character, and therefore little affinity towards the environment and low swelling capacity. The present study suggests that this protection of the material by the CH_3_ groups is a predominant factor concerning the size of the molecular chain. Furthermore, after 24 h, all membranes reached the plateau.

Additionally, it is worth stating that these results are essential when seeking to develop membranes with a low inflammatory response since high values of swelling (>15%) increase the immune response, as observed in the in vivo studies [14].

### 3.3. Small-Angle X-ray Scattering (SAXS)

**Figure 3:**
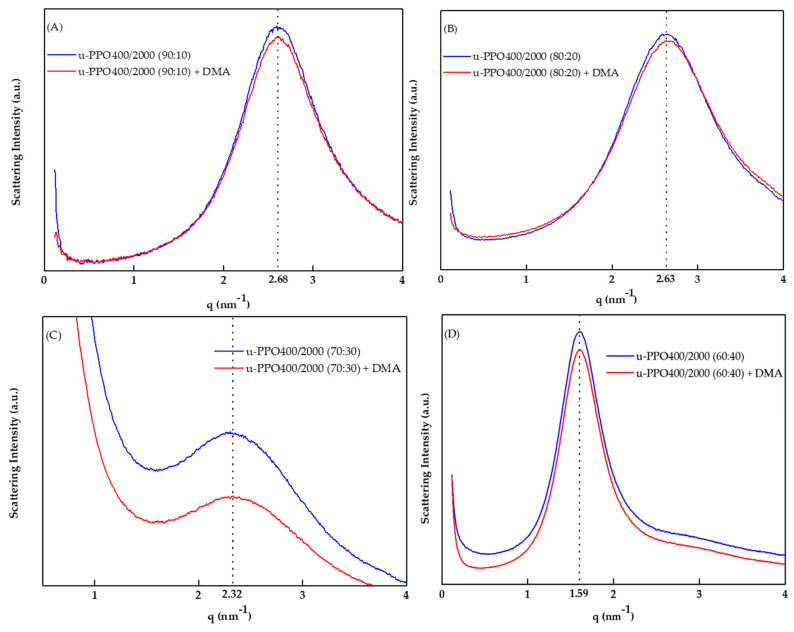
SAXS curves for ureasil–polyether membranes with and without dexamethasone. (**A**) u-PPO400/2000 (90:10), (**B**) u-PPO400/2000 (80:20), (**C**) u-PPO400/2000 (70:30), and (**D**) u-PPO400/2000 (60:40). All the red curves represent the membranes with dexamethasone and the blue curves represent the membranes without dexamethasone. Abbreviation: DMA = dexamethasone.

The small-angle X-ray scattering (SAXS) technique enabled the observation of the effects of the incorporation of DMA into the ureasil–polyether membrane nanostructure. Figure 3 shows the SAXS curves corresponding to the u-PPO400/2000 membranes with and without DMA at 3%.

The presence of a well-defined peak demonstrates the spatial correlation between the silicon nodes of the polymeric network, revealing the structural homogeneity of the matrix [21]; the values from the maximum peak of the scattering vector (*q*_max_) were 2.68, 2.63, 2.32, and 1.59. Thus, the distances between the silicon nodes (*d*) were calculated by the equation d=2π/qmax to be 2.34, 2.38, 2.70, and 3.94 nm for the samples u-PPO400/2000 90:10, u-PPO400/2000 80:20, u-PPO400/2000 70:30, and u-PPO400/2000 60:40, respectively. It is essential to note that the incorporation of DMA did not shift the *q*_max_ value. However, the values of *d* changed (an increase in the distance) as the proportion of u-PPO2000 precursor increased in the sample, which is attributed to the higher molecular mass of this polymer [22]. Importantly, the intensity of *q*_max_ decreased for all samples when DMA was not used, indicating the decrease in electron density between the silicon nodes (Si-O-Si); this event indicates that DMA molecules prefer the organic phase of the material.

### 3.4. In Vitro DMA Release

A linear regression was performed on the analytical curve (Figure S1) and the linear equation was: *y* = 0.0381 *x* + 0.0917, with the determination coefficient *R*^2^ = 0.9985, indicating a significant linear regression (*p* < 0.0001) and an adequate linearity for the concentrations in the range from 1 to 50 µg/mL.

**Figure 4:**
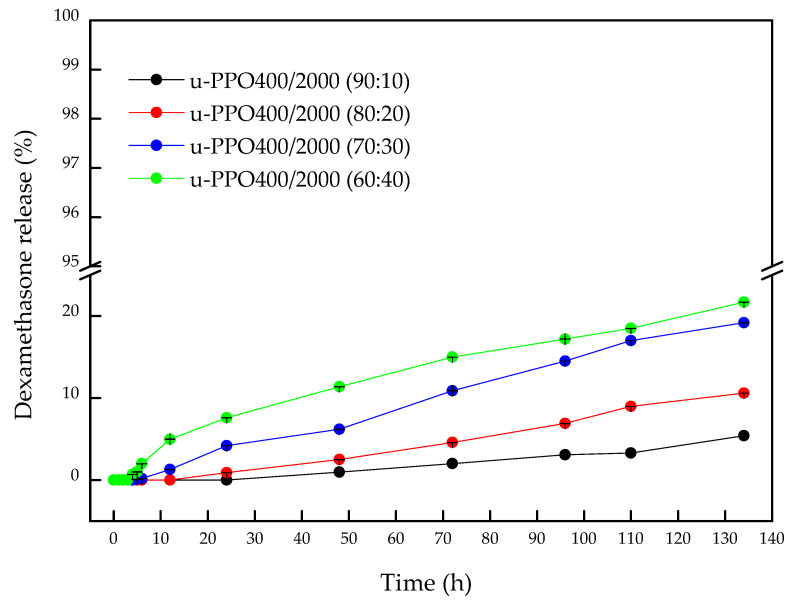
Release profile of dexamethasone incorporated in the u-PPO400/2000 membranes in different proportions: 90:10, 80:20, 70:30, and 60:40. Results are expressed as the mean ± SD of triplicates (*n* = 3).

Figure 4 shows the release profiles of API from the u-POP400/2000 membranes at different proportions.

The results demonstrated that all membranes, regardless of the proportion, did not present a Burst effect; thus, a low amount of DMA was released throughout the experiment (<22% during 134 h). Table S1 presents all values of the percentage of DMA released from the membranes for better visualization. These results present the possibility of controlling the release for days or even months since a plateau was not observed. The u-PPO400/2000 (90:10), u-PPO400/2000 (80:20), u-PPO400/2000 (70:30), and u-PPO400/2000 (60:40) membranes initiated the release process after 24, 12, 4 and 3 h, respectively, with a delayed and prolonged release profile. The minimum deviation values indicated the reproducibility of the results, which were correlated with SAXS, demonstrating the homogeneity of the nanostructure of the materials. Statistically, the u-PPO400/2000 (90:10) material showed no difference in its release profile among the times of 48, 72, 96, and 110 h, suggesting that it reached the plateau; however, after 134 h, a significant difference occurred (*p* ≤ 0.001) releasing 5.4% of DMA.

Regarding u-PPO400/2000 (80:20), it was observed that after 96, 110, and 134 h, it showed a significant difference with *p* ≤ 0.01, *p* ≤ 0.001, and *p* ≤ 0.001, with the release percentage of 6.9, 9, and 10.6%, respectively. While, for u-PPO400/2000 (70:30), the release time that showed a significant difference was 72 h (*p* ≤ 0.001), reaching 6.2% of release and, at 134 h, 19.20%. An interesting fact was observed when comparing the results between u-PPO400/2000 (70:30) and u-PPO400/2000 (60:40), since, statistically, after 72 h, all release values showed no statistical difference.

The increase in the release due to the higher amounts of u-PPO2000 precursor can be explained by two factors: first, its hydrophobic character, as the u-PPO400 has a lower molecular weight compared to u-PPO2000, explaining the low affinity with the environment; second, the membranes with a higher proportion of u-PPO400 material present higher rigidity (many siloxane groups), decreasing the ability of relaxation and swelling, as observed in the results of swelling [23,24,25,26,27], explaining the greater or lesser ability of the material to swell and the process of diffusion of the environment to occur, a fact observed in the difference in detection time of DMA.

In the literature, few studies have demonstrated DMA-encapsulated membranes with delayed and prolonged release capabilities. Poly (lactic-co-glycolic acid) (PLGA) scaffold-type membranes showed an in vitro release of 90% at day 14 [24]. Another study using nanofibers revealed a cumulative release for 23 days. However, unlike the results obtained in this study, ~56% of DMA was released after 24 h [25].

Finally, different mathematical models were applied to release data, such as Korsmeyer–Peppas, Higuchi, Hixson–Crowell, Baker–Lonsdale, zero order, and first order. However, the mathematical model described by Korsmeyer and Peppas (Equation (2)) had the best fit for the experimental release curves (*r*^2^).

(2) $$\frac{Mt}{M\infty }{=K\;\times \;t}^{n}$$

From Equation (2), *Mt* is the amount of DMA released per area in a time t; *M∞* is the total amount of DMA released in an infinite time; k is the release constant that considers structural and geometric characteristics of the systems; and *n* is the exponent value that defines the release mechanism.

Thus, assuming a cylindrical shape, the values of the *n* parameter (Table S2) were above 0.88 for all curves, corresponding to the transport mechanism of case II, where the difference concerning the anomalous transport is caused only by the order of velocity of the release processes (diffusion and swelling). Therefore, the DMA diffusion velocity is higher with the relaxation of the material chains, which contributes to the prolonged release observed in this study [28,29]. Macroscopic swelling and drug release test results allowed us to analyze and rationally select the membrane u-PPO400/2000 (70:30) for the following experiments.

### 3.5. Atomic Force Microscopy (AFM)

AFM experiments were performed to better understand the surface of these materials since roughness is essential for the adhesion of different proteins and cell membrane receptors, which can result in the increased attraction of osteoblasts for extracellular matrix formation over the entire surface of the material [30].

**Figure 5:**
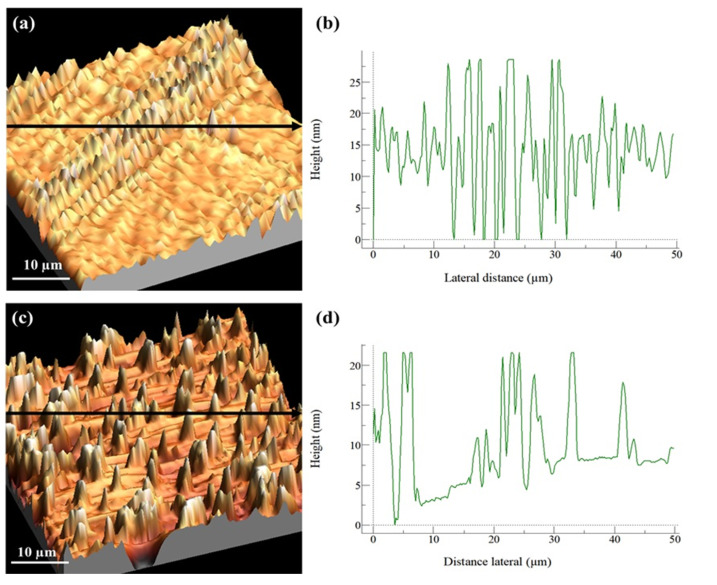
(**a**,**b**) u-PPO400/2000 membrane (70:30% *w*/*w*) without DMA. (**a**) Three-dimensional topography in tapping-mode AFM. (**b**) Contour along the arrow in (**a**). (**c**,**d**) u-PPO400/2000 membrane (70:30% *w*/*w*) with DMA. (**c**) Three-dimensional topography in contact-mode AFM. (**d**) Contour along the arrow in (**c**).

Figure 5 shows the 3D topography results for the u-PPO400/2000 membrane (70:30% *w*/*w*) with and without DMA.

It was observed that for the u-PPO400/2000 membrane (70:30% *w*/*w*) without DMA, a flat surface was revealed, with an average roughness of ~5.9 nm and a central region (Figure 5a) with maximum height values of 28.58 nm, demonstrating that the surface of these materials was flat. However, we can observe that the surface roughness was higher than that of the u-PPO400/2000 membrane (70:30% *w*/*w*) with DMA. Thus, the addition of DMA did not result in a significant change in the average roughness; however, two types of structural organization on the surface were observed.

Figure 5a has a concentrated region with 15 µm peak maximum values, while Figure 5c shows a homogeneous distribution of these peaks with maximum values over the entire surface, in addition to the large part of the surface presenting a flat/smooth surface. The addition of DMA (hydrophobic) may have altered and decreased the average roughness due to hydrophobic/hydrophobic interactions or weak bonding between these materials, resulting in the different homogeneity of the peaks observed. No pores and/or crystalline precipitates were observed corresponding to DMA; thus, it is possible to conclude that the added ratio did not precipitate, as seen in Figure 2.

### 3.6. Evaluation of the Hemolytic Potential of Ureasil–Polyether Materials

**Figure 6:**
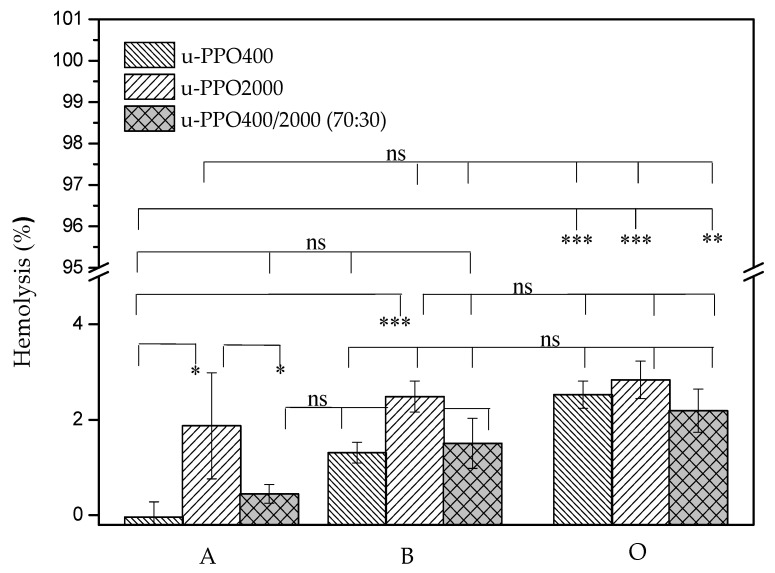
Percentage of hemolytic potential between u-PPO400 and u-PPO400/PPO2000 (70:30) against blood types A, B, and O. Results are expressed as the mean ± SD of triplicates (*n* = 3). * *p* < 0.05, ** *p* < 0.01, *** *p* < 0.001, ns: no significant difference.

Once a device composed of biomaterials or a medical device comes into contact with blood, several mechanisms and reactions are triggered, so it is extremely essential to have a test that proves the safety of its application. Hemolysis represents the breakdown of the integrity of the red cell membrane (erythrocytes). Therefore, a hemolysis test allows the evaluation of the integrity of these cells after they come in contact with the biomaterial. When the results show a percentage greater than 5% after contact with the device studied, this is considered a hemolytic material, according to ASTMF756-00:2000—Standard Practice for Assessment of Hemolytic Properties of Materials from the American Society for Testing and Materials [31,32]. Therefore, Figure 6 shows the hemolytic potential results for the u-PPO400 and u-PPO400/PPO2000 (70:30) samples.

We can observe that the u-PPO2000 membrane revealed the highest values of hemolysis when compared to u-PPO400, being 1.87 ± 1.11 and 2.48 ± 0.32 for blood type A and B, respectively. However, both showed no significant difference in the presence of blood type O. Upon analysis of the u-PPO400/2000 mixture, we can observe that it did not present a statistical difference between blood groups A with B and B with O, it only had a difference between groups A and O. Despite these considerations, from the point of view of biocompatibility of the material, all groups were considered to be within the hemocompatibility range. Furthermore, we can observe that all tested materials presented hemocompatibility, regardless of the blood type used. The positive control showed 100% cell lysis, while the negative control showed no lysis (data not shown). These data are in accordance with other studies in the literature, which demonstrate by cytotoxicity assay in keratinocytes cells that these u-PPO type materials are biocompatible [33].

### 3.7. In Vivo Assay

**Figure 7:**
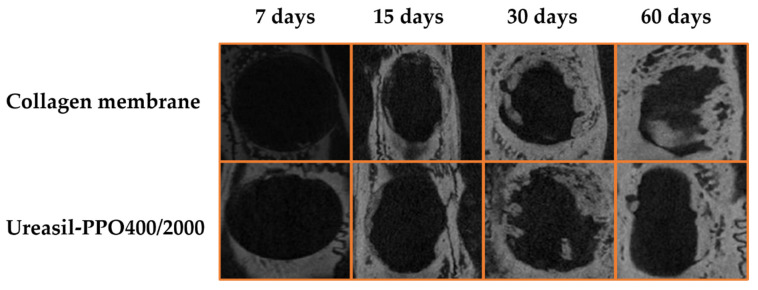
Photomicrotomography of the bone defects containing the commercial membrane and u-POP400/2000 membrane.

The in vivo assay was performed comparing a commercial collagen membrane and the u-PPO400/2000 membrane (70:30); this proportion was the only one adopted, aiming to meet the principle of the 3Rs—reduce, reuse, and recycle. Thus, based on the swelling and release results, this proportion was chosen. Figure 7 presents the photomicrographs of the bone defects in the cranial skull at 7, 15, 30, and 60 days for the bone defects with the collagen membrane and the u-POP400/2000 membrane.

The photomicrotomographs revealed that a gradual increase of bone tissue was being formed despite the material used, and in both cases total bone healing was not achieved. In order to verify the bone volume formed, CTAn software was used. After delimiting the region of interest (ROI) in 2D, the software presents the results in 3D through the collective sum of all ROIs from a set of transversal image slices. Table 2 presents the percentage volume and values of the bone tissue volume formed.

Bone defects in the initial period (7 days) showed a statistically (*p* < 0.001) lower formation for the u-PPO400/2000 membrane compared to the collagen group. However, statistically, the same increase in the percentage of bone volume formed was observed on days 15 and 30, with ~12 and 20% (*p* < 0.001). Following that, no significant difference was observed at 60 days (~21%). With longer observation periods, the bone volume would probably not be higher. The bone defects in this study are of a critical type so that the body is dependent on the help of a material or device. However, we can confirm the physical barrier property of the developed membrane and its ability to incorporate and release active substances, aiming at complete tissue regeneration without the need for grafting techniques, as observed in works with hydroxyapatite coated with an ε-polycaprolactone polymer emulsion incorporated with bone morphogenetic protein 2 nanoparticles (PCL_BMP-2/NPs) [34] and the polyurethane/hydroxyapatite-based polymeric foam [35].

## 4. Conclusions

Hydrophobic membranes were developed using proportions of two hybrid precursors, u-PPO400 and u-PPO2000. The results demonstrated that the increase of the proportion of u-PPO2000 material in the membranes modified the swelling profile and release of the model drug dexamethasone, a fact tied to their high molecular mass. We highlighted the low swelling (>7%) of all proportions, which prevent tissue damage when implanted and the delayed and prolonged controlled release capacity (no plateau and <22% after 5 days), which is a result not commonly found in other materials in the literature and, enables the treatment for several days, taking into consideration that bone defects of critical size take months to heal. Finally, the physical barrier capacity was compared with a commercial membrane, statistically revealing the same volume of bone formed. The hydrophobic membranes, despite their significant potential, must be tested with growth factors, therefore the next step will be to conduct phase I studies, since the low production cost could benefit both patients and the health system in general.

## Figures and Tables

**Table 1:** Percentage of swelling of membranes from the mixtures of ureasil–polyether precursors in the proportions of 90:10; 80:20; 70:30 and 60:40, immersed in phosphate buffer at pH 7.2.

*Time* (*min*)	*Swelling* (*%*)			
	* *Ureasil*– *PPO400*/*PPO2000* *90:10*	* *Ureasil*– *PPO400*/*PPO2000* 80:20	* *Ureasil*– *PPO400*/*PPO2000* *70:30*	* *Ureasil*– *PPO400*/*PPO2000* *60:40*
0	0.00	0.00	0.00	0.00
15 *	0.43 ± 0.02	0.67 ± 0.02	070 ± 0.06	1.38 ± 0.12
30 *	1.17 ± 0.09	2.25 ± 0.1	2.12 ± 0.31	1.62 ± 0.26
60 *	2.43 ± 0.22	2.46 ± 0.12	2.51 ± 0.22	1.96 ± 0.13
90 *	2.63 ± 0.07	2.88 ± 0.44	3.00 ± 0.38	3.52 ± 0.44
120 *	2.67 ± 0.17	2.76 ± 0.11	3.64 ± 0.25	4.61 ± 0.36
360 *	2.69 ± 0.11	2.90 ± 0.16	4.24 ± 0.24	4.69 ± 0.33
720 *	3.35 ± 0.01	3.72 ± 0.18	4.40 ± 0.18	5.05 ± 0.57
1440 *	4.10 ± 0.11	4.30 ± 0.14	4.53 ± 0.17	5.99 ± 0.31
2880 *	4.25 ± 0.15	4.55 ± 0.14	4.65 ± 0.11	6.24 ± 0.66
4320 *	4.32 ± 0.11	4.58 ± 0.15	5.39 ± 0.22	6.88 ± 0.49

Results are expressed as the mean ± SD of triplicates (*n* = 3). * Statistically significant difference among means (*p* < 0.001).

**Table 2:** Percentage of bone tissue volume in the critical-sized bone defect at 7, 15, 30, and 60 days after the surgical procedure. Values expressed in percentage of volume and mean ± SD.

Membranes Types	7 Days	15 Days	30 Days	60 Days
Commercial (collagen)	7 ± 0.73 ^a^	12.66 ± 0.63 ^c^	19.66 ± 1.43 ^d^	21.9 ± 0.28 ^d^
u-PPO400/PEO1900	4 ± 0.63 ^b^	12.18 ± 1.2 ^c^	20.28 ± 0.30 ^d^	21.1 ± 0.08 ^d^

^a–d^ Different letters in each column indicate that the differences between means were statistically significant (*p* < 0.05).

## Data Availability

Link to publicly archived datasets analyzed or generated during the study. Please refer to suggested Data Availability. https://1drv.ms/u/s!AnTvCVJp6xpjiKk8qbbSH3J2h7TD6A?e=b0qsRj (accessed on 27 March 2022).

## Supplementary Materials

The following supporting information can be downloaded at: https://www.mdpi.com/article/10.3390/pharmaceutics14051027/s1, Figure S1: Linear regression of dexamethasone. Table S1. Percentage of release to membranes from ureasyl-polyether precursor mixtures: 90:10; 80:20; 70:30, and 60:40. Results are expressed as the mean ± SD of triplicates (*n* = 3). Table S2. Korsmeyer-Peppas equation parameters for the different dexamethasone release curves for u-PPO400/2000 membranes at the proportions 90:10, 80:20, 70:30, and 60:40.
