# Supplementary material for: Dexamethasone-Loaded Ureasil Hydrophobic Membrane for Bone Guided Regeneration
Source: Pharmaceutics. 2022 May 10;14(5):1027. doi: 10.3390/pharmaceutics14051027 (PMC9146579; doi:10.3390/pharmaceutics14051027)
Supplement: Supplementary file 1 [file pharmaceutics-14-01027-s001.zip › pharmaceutics-1677391-supplementary.pdf]

pharmaceutics

MDPI

Supplementary Materials: Dexamethasone Loaded-Ureasil Hydrophobic Membrane for Bone Guided Regeneration

Rafaella Barros, Camila Garcia da Silva, Kammila Martins Nicolau Costa, Arnobio A. da Silva-Junior, Cássio Rocha Scardueli, Rosemary Adriana Chierici Marcantonio, Leila Aparecida Chiavacci and João Augusto Oshiro-Junior

Citation: Barros, R.M.; da Silva, C.G.; Nicolau Costa, K.M.; da Silva-Junior, A.A.; Scardueli, C.R.; Marcantonio, R.A.C.; Chiavacci, L.A.; Oshiro-Junior, J.A. Dexamethasone-Loaded Ureasil Hydrophobic Membrane for Bone Guided Regeneration. *Pharmaceutics* **2022**, *14*, 1027. https://doi.org/10.3390/pharmaceutics14051027

Academic Editors: Isabel Izquierdo-Barba and Marina Gallarate

Received: 28 March 2022
Accepted: 9 May 2022
Published: 10 May 2022

Publisher's Note: MDPI stays neutral with regard to jurisdictional claims in published maps and institutional affiliations.

Copyright: © 2022 by the authors. Licensee MDPI, Basel, Switzerland. This article is an open access article distributed under the terms and conditions of the Creative Commons Attribution (CC BY) license (https://creativecommons.org/licenses/by/4.0/).

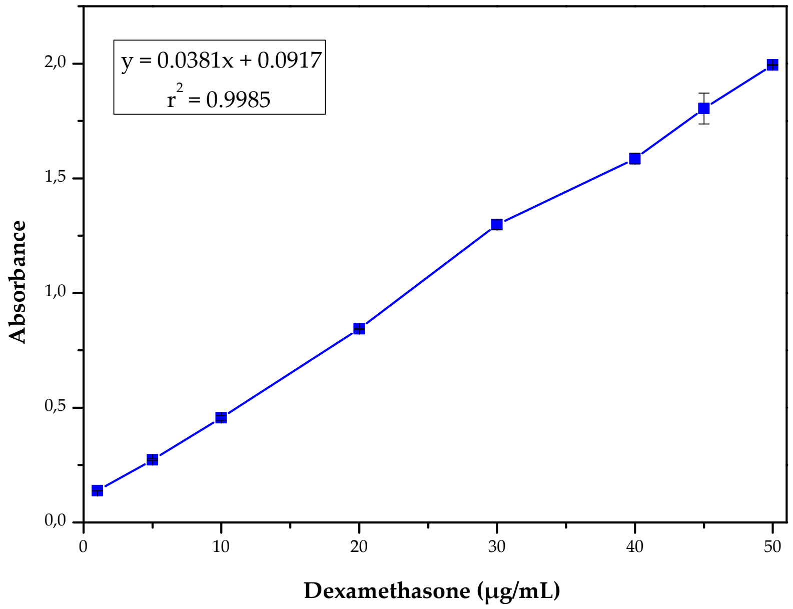

Figure S1. Linear regression of dexamethasone.

Table S1. Percentage of release to membranes from ureasyl-polyether precursor mixtures: 90:10; 80:20; 70:30, and 60:40. Results are expressed as the mean ± SD of triplicates (n = 3).

**Time (h)**	**Release (%)**			
	**u-PPO400/2000 90:10**	**u-PPO400/2000 80:20**	**u-PPO400/2000 70:30**	**u-PPO400/2000 60:40**
0	0.00	0.00	0.00	0.00
1	0.00	0.00	0.00	0.00
2	0.00	0.00	0.00	0.00
3	0.00	0.00	0.00	0.00
4	0.00	0.00	0.00	0.70 ± 0.0034
5	0.00	0.00	0.10 ± 0.0087	1.00 ± 0.00935
6	0.00	0.00	0.24 ± 0.00617	2.3 ± 0.00112
12	0.00	0.00	1.30 ± 0.00351	5.05 ± 0.00586
24	0.00	0.90 ± 0.0015	4.20 ± 0.005	7.60 ± 0.00418
48	0.98 ± 0.0033	2.50 ± 0.0059	6.20 ± 0.0024	11.40 ± 0.0056
72	2.00 ± 0.0033	4.60 ± 0.0046	10.90 ± 0.0024	15.00 ± 0.00252
96	3.10 ± 0.0044	6.90 ± 0.0024	14.50 ± 0.00733	17.2 ± 0.00833
110	3.30 ± 0.003	9.00 ± 0.004	17.00 ± 0.0033	18.5 ± 0.00762
134	5.40 ± 0.004	10.6 ± 0.0056	19.20 ± 0.00651	21.7 ± 0.01184

Table S2. Korsmeyer-Peppas equation parameters for the different dexamethasone release curves for u-PPO400/2000 membranes at the proportions 90:10, 80:20, 70:30, and 60:40.

**u-PPO400/2000 Proportion**	**r^2 adjusted**	**Equation Parameters**		**Release mechanism**
		**K**	***n***	
(90:10)	0.9953	0.0011	1.72	Case II
(80:20)	0.9978	0.0117	1.39	Case II
(70:30)	0.9941	0.1259	1.034	Case II
(60:40)	0.9936	0.6836	0.8888	Case II
